# Transcription factors ETV4 and ETV5 are required for nephron progenitor cell maintenance, distal nephron development and connection to the collecting system

**DOI:** 10.3389/fcell.2026.1820140

**Published:** 2026-07-01

**Authors:** Kaitlin Day, Konrad Thorner, Milanya Thumma, Yanzhen Zhang, Evan VordemEsche, William Kuhlman, Aiden Varnell, Anhar Hosawi, Benjamin Hammond, Madison Fraunfelter, Prashant Gaikwad, Matt Kofron, Franklin D. Costantini, Cristina Cebrian

**Affiliations:** 1 Molecular and Developmental Biology Graduate Program, Cincinnati Children’s Hospital Medical Center, Cincinnati, OH, United States; 2 Department of Pediatrics, University of Cincinnati College of Medicine, Cincinnati, OH, United States; 3 Division of Developmental Biology, Cincinnati Children’s Hospital Medical Center, Cincinnati, OH, United States; 4 Department of Genetics and Development, Columbia University, New York City, NY, United States

**Keywords:** cysts, embryo, Etv4, Etv5, kidney development, nephrogenesis, nephron progenitor cell (NPC)

## Abstract

The functional unit of the kidney, the nephron, is composed of the renal corpuscle and the renal tubule that includes proximal tubule, loop of Henle, distal and connecting tubule. Each of these segments perform distinct and complementary roles to ensure body homeostasis and urine excretion into the renal collecting system. During nephrogenesis, nephron progenitor cells (NPCs) either self-renew to maintain the progenitor pool or condense and epithelialize into nascent nephrons. Two transcription factors, ETV4 and ETV5 are downstream targets of receptor tyrosine kinase signaling and are known to act redundantly to drive development of the renal collecting system, but their possible role in nephrogenesis has not been elucidated. Here, we show that *Etv4* and *Etv5* are expressed in NPCs and developing nephrons. Ablation of *Etv5* from NPCs in an *Etv4* null background results in hypoplastic kidneys, ectopic NPC differentiation, premature NPC depletion and a severely cystic phenotype. The majority of cysts in these mutants originate from the S3 proximal tubule segment; however, removing *Etv4* and *Etv5* from the proximal developing nephron was insufficient to recapitulate the cystic phenotype. Further analysis of the NPC double mutant kidneys revealed a significant decrease in distal nephron markers and failure to connect to the collecting duct system in mutant embryonic kidney explants. These findings reveal novel roles for ETV4 and ETV5 in nephrogenesis, from progenitor maintenance to nephron segmentation.

## 1 Introduction

Kidney organogenesis in mice begins around embryonic day 10.5, when the ureteric bud invades the metanephric mesenchyme initiating a series of reciprocal interactions driving ureteric bud branching and nephrogenesis (Saxen, 1987; Costantini and Kopan, 2010). This process continues until around postnatal day 2, when the nephron progenitor cells (NPCs) within the metanephric mesenchyme undergo differentiation en masse (Hartman et al., 2007). Therefore, throughout kidney development, the NPCs experience a delicate balance between self-renewal, to ensure the availability of progenitors throughout kidney development, and differentiation, to give rise to enough nephrons for proper kidney function. The rate of nephron induction is slower early in development and peaks towards the end of nephrogenesis (Cebrián et al., 2004; Short et al., 2014); hence, ensuring that enough progenitors reach that later stage is critical to generate the full nephron complement. Several key transcription factors (SALL1, OSR1, SIX2, PAX2) signaling pathways (WNT/B-catenin, FGFs, BMPs) and epigenetic regulators (PRC2 complex, BRG1, HDACs) are known to modulate self-renewal and/or differentiation of NPCs, and alterations in these genes/pathways lead to complete renal agenesis or severe hypoplasia (Dudley et al., 1995; Luo et al., 1995; Torres et al., 1995; Carroll et al., 2005; Wang et al., 2005; Self et al., 2006; Blank et al., 2009; Brown et al., 2011; Barak et al., 2012; Basta et al., 2014; Basta et al., 2020; Xu et al., 2014; Naiman et al., 2017; Liu et al., 2018; Liu et al., 2020).

Throughout kidney development, these self-renewing NPCs also differentiate to generate nephrons. Nephrogenesis involves a network of complex morphological stages and signaling events: NPCs differentiate first into a pretubular aggregate before epithelializing into a renal vesicle, and developing into a comma-shaped body, and S-shaped body before finally developing into a mature nephron. The nephron is the functional unit of the kidney, generating a rough filtrate from the blood that is refined through water and nutrient reabsorption as it moves through each segment of the nephron. Importantly, for this filtrate to be released as urine, the nephrons must connect to the ureteric bud-derived collecting system. This connection is not present immediately. The renal vesicle maintains contact with the ureteric bud, but it is not till the S-shape body stage that a continuous lumen is established between the developing nephron and the collecting system (Kao et al., 2012). Adherence junctions (Yang et al., 2013) and HGF-Met signaling (López-García et al., 2024) have been shown to mediate the establishment of a continuous lumen, although the cellular and molecular mechanisms directing the connection of the nephron to the collecting system are not fully characterized.

ETV4 and ETV5 (from now on, ETV4/5) are members of the PEA3 family of ETS transcription factors. They play important roles in limb, lung and neural development (Ladle and Frank, 2002; Livet et al., 2002; Mao et al., 2009; Zhang et al., 2009; Herriges et al., 2015) by regulating cell proliferation, differentiation and migration. In humans, mutations in *ETV4* have been identified in two independent cases of congenital anomalies of the kidney and urinary tract (Chen et al., 2016; Kolvenbach et al., 2023). In the developing mouse lung, they mediate FGF10 signaling to promote *Shh* expression that would in turn inhibit FGF signaling, hence part of a negative feedback loop (Herriges et al., 2015). By contrast, in the mouse developing limb, *Etv4/5* are also downstream of FGF signaling, but they act by downregulating *Shh* (Mao et al., 2009; Zhang et al., 2009). In the developing kidney, they were identified as major downstream targets and effectors of RET-GDNF signaling; they are expressed at the tip of the ureteric bud and act redundantly to drive ureteric bud formation (Kuure et al., 2010) and branching (Lu et al., 2009) by regulating directed cell movements (Riccio et al., 2016).

In addition to their expression in the ureteric bud, *Etv4/5* are also expressed in the metanephric mesenchyme and early developing nephrons although their role in these compartments has not been elucidated. *Etv4/5* expression is upregulated by FGFR signaling in the metanephric mesenchyme (Brown et al., 2011; Barak et al., 2012) and lost when FGFR signaling is reduced (Barak et al., 2012) suggesting a role as major downstream targets of FGF signaling in the mesenchyme. To investigate the role of Etv4/5 in nephrogenesis we have ablated *Etv5* in NPCs in an *Etv4* null background mouse. Here we report that absence of these transcription factors leads to premature exhaustion of the progenitors, as well as failure of the developing nephrons to establish a connection with the collecting ducts, and a severely cystic phenotype.

## 2 Materials and methods

### 2.1 Mice

Mouse experiments were performed in accordance to animal care guidelines and approved by the Institutional Animal Care and Use Committee of Cincinnati Children’s Hospital Medical Center (IACUC 2023-1021). The following mouse alleles have been previously published and were used and maintained on a mixed background: Six2^(EGFP/cre)1Amc^ (*Six2-creEGFP*) (Kobayashi et al., 2008), Osr2^tm2(cre)Jian^ (*Osr2-IresCre*) (Lan et al., 2007), *Etv4* ^*tm1Arbr*^ *(Etv4-)* (Livet et al., 2002), and *Etv5* ^*tm1.1Xsun*^ (*Etv5* ^*Fl*^) (Zhang et al., 2009). For embryonic samples, the day the vaginal plug was observed was considered as embryonic day (E) 0.5. Genotyping was performed using either ear or tail clippings.

### 2.2 Histology

Kidneys were dissected in 1xPhosphate-Buffered Saline (PBS) and processed for either paraffin or OCT embedding. Paraffin samples were fixed at 4 °C in 10% neutral buffered formalin overnight, washed in 1xPBS and paraffin embedded by the CCHMC Integrated Pathology Research Facility. 8um sections taken and placed on slides before baking slides overnight at 50 °C and used for standard Hematoxylin/Eosin (H&E) staining and immunofluorescent staining. For cryosectioning, kidneys were fixed at 4 °C in 4% Paraformaldehyde for 30 min. After washing with 1xPBS three times, the kidneys were placed in 30% sucrose overnight at 4 °C then mounted in O.C.T. and stored at −80 °C. 8um sections were taken on an Epredia CryostarNX70 cryostat and placed on Superfrost slides for immunofluorescent staining.

### 2.3 Explant cultures

Kidneys were dissected at E12.5 and cultured at 37 °C in 5%CO_2_ for 3 days on 0.4 um polyester membranes (Corning→ Transwell→ 3450) using explant culture medium (DMEM-F12, 10% Fetal Bovine Serum, 1xGlutamax and Penicillin-Streptomycin). For the timelapse imaging of these cultures we used a Nikon Ti2 inverted SpectraX wide-field fluorescent microscope with an incubation chamber (Costantini et al., 2011). Both brightfield and endogenous fluorescence was imaged every 30 min during the incubation period. After culture, kidneys were fixed in 4% PFA for 10 min at room temperature and washed three times in PBS for use in immunofluorescent staining.

### 2.4 Immunofluorescent staining

Paraffin slides were placed in Trilogy Antigen Retrieval (Cell Marque, 920P-10) and boiled in a pressure cooker on high pressure for 15 min. Slides were then moved to a hot Trilogy bath for 5 min before being washed three times in 1xPBS before immunofluorescent staining. OCT slides were washed three times in 1xPBS before immunofluorescent staining. Prepared slides were first placed in a blocking solution [PBST, (0.1% Triton X-100 in 1xPBS) and 10% donkey serum] for 1 h at room temperature. Slides were incubated in a primary antibody in PBST for either 1 h at 37 °C or overnight at 4 °C. After three PBS washes, the slides were then incubated with secondary antibody in PBST for either 1 h at 37 °C or 3 h at room temperature. Slides were then washed in PBS three times and mounted in Prolong Gold Antifade Reagent (Cell Signaling Technology, 9071S).

Fixed kidney explants were cut from the main filter and placed in a 24-well plate to be incubated in primary antibody solution in TSP (0.1% v/v Triton-X100, 0.05% w/v Saponin in 1xPBS) overnight at 4 °C. After three PBS washes, the slides were then incubated with secondary antibody in TSP overnight at 4 °C. The explants were then washed in PBS three times and mounted in Prolong Gold Antifade Reagent (Cell Signaling Technology, 9071S). Primary antibodies/lectins used: AQP1 (Proteintech 20333-1-AP), Calbindin1 (Proteintech 14479-1-AP), DBA (Vector Laboratories B-1035-5), ETV4 (Proteintech 10684-1-AP), ETV5 (Proteintech 13011-1-AP), HNF1B (Proteintech 12533-1-AP), KRT8 (DSHB, TROMA-1), LTL (Vector Laboratories B-1325-2), PDGFR**α** (R&D Systems AF1062), SALL1 (Abcam ab41974), SLC12A1 (Proteintech 18970-1-AP), SLC12A3 (Thermo Fisher PA5-77816), TFAP2B (Proteintech 13183-1-AP) and ZO1 (Thermo Scientific 33-9100).

### 2.5 Imaging and quantification

All H&E imaging was done on a Nikon NiE wide-field upright microscope. Immunofluorescent imaging was done on either a Nikon AXR inverted or a Yokogawa SoRa W1 confocal microscopes. Analysis was done using Nikon Elements software. For segment area quantification, a threshold was defined for each stain before a custom GA3 workflow was used to measure total staining area for each marker (Method modified from (Duvall et al., 2022)). Each marker analyzed is represented as percentage of total surface. Thresholding of tissue surface is required to avoid overestimation of total surface in cystic samples. See Supplementary Figure 2.

### 2.6 RNAscope

Detection of *Wnt4* mRNA was performed on thin paraffin sections using RNAscope Multiplex Fluorescent Reagent Kit V2 and RNAscope Probe-Mm-Wnt4-C2 following manufacturer’s instructions. Sections were imaged on a Nikon AXR inverted confocal microscope.

### 2.7 RNA extraction, sequencing and analysis

Both kidneys from *Etv4/5* mutant embryos and control littermates were isolated at E14.5 and snap frozen using dry ice. RNA was extracted from 4 controls and 4 experimental kidney pairs using the Nucleospin RNA prep kit (Takara Bio, 740984.50) and submitted to Novogene for library preparation and paired-end sequencing. Initial RNAs and library passed all quality controls. However, one of the control samples failed quality control upon sequencing and those results were excluded from the subsequent analysis.

Raw data was processed with fastp (version 0.23.2) for initial trimming and quality control. Pseudoalignment was performed with kallisto (version 0.46.0) against the mm10 transcriptome. DESeq2 (version 1.40.2) was used for differential expression and visualization. Sex-regulated genes were not included to avoid bias caused by sex imbalance in original samples. Genes with Log2 fold changes greater than +/- 0.5 with padj values smaller than 0.05 were considered differentially expressed and are listed in Supplementary Table S1.

## 3 Results

### 3.1 ETV4/5 are required in both the ureteric bud and the NPCs, and their ablation causes severe renal defects

**FIGURE 1:**
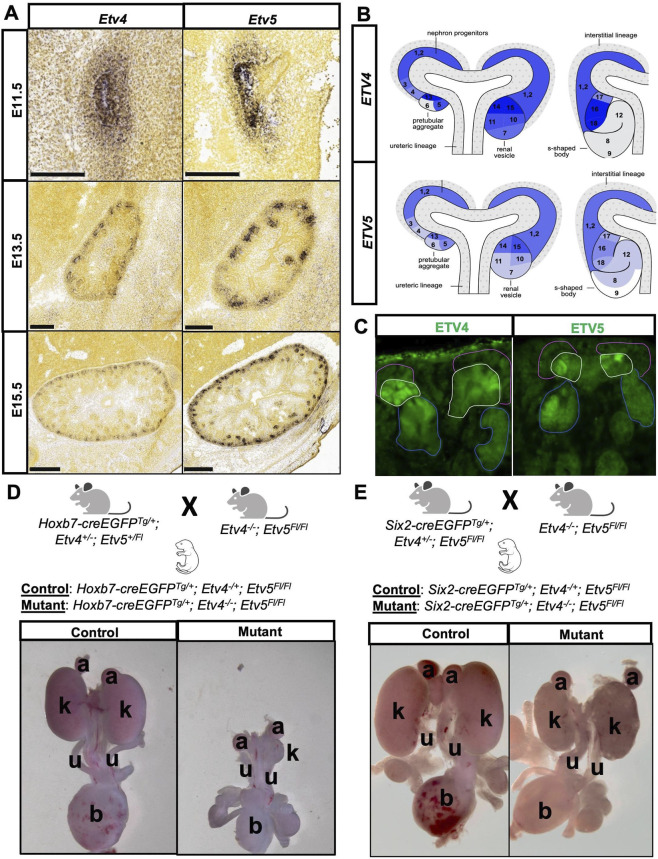
Expression of *Etv4* and *Etv5* in the developing kidney and consequences of *Etv4*/*5* ablation. **(A)** *In Situ* Hybridization assay showing that *Etv4* and *Etv5* are expressed in the tips of the UB, the NPCs and the developing nephrons. Allen Mouse Brain Atlas, https://developingmouse.brain-map.org/experiment/show/100056741 (Etv4, E11.5), /100055444 (Etv5, E11.5), /100057648 (Etv4, E13.5), /100076595 (Etv5, E13.5), /100057668 (Etv4, E15.5) and /100083513 (Etv5, E15.5). Scale bar, 500 um. **(B)** In humans, *ETV4* and *ETV5* are expressed most prominently in NPCs, PTAs, RVs and the distal portion of developing nephrons, scRNA-seq data obtained from The Human Nephrogenesis Atlas. **(C)** Immunofluorescent stain of ETV4 and ETV5 in UB tips (white lines), the Cap mesenchyme (pink lines) and the developing nephrons (blue lines) in P0 murine kidneys. **(D)** Mating strategy for a *Hoxb7-cre* that removes ETV4 and ETV5 from the collecting duct system. The resulting mutants exhibit kidney phenotypes ranging from severe hypoplastic kidneys to renal agenesis [a = adrenal gland, k = kidney, u = ureter, b = bladder]. **(E)** Mating strategy for a *Six2-cre* line that removes ETV4 and ETV5 from NPC’s and NPC-derived cells. The resulting mutants develop cystic, hypoplastic kidneys [a = adrenal gland, k = kidney, u = ureter, b = bladder].

To elucidate the roles of ETV4/5 in kidney development, we first investigated the spatial expression of the two transcription factors in the developing mouse kidney. *In situ* hybridization data from the Allen Brain Atlas (Allen Institute for Brain Science, 2008. Allen Mouse Brain Atlas [Developing Mouse Brain]. Available from mouse.brain-map.org, n.d.) indicate expression in the ureteric bud tips and mesenchyme at embryonic day 11.5 (E11.5), and in the ureteric bud tips, mesenchyme and developing nephrons at E13.5 and E15.5 (Figure 1A). This mesenchymal expression pattern is also observed in human scRNA-seq data from The Human Nephrogenesis Atlas (Lindström et al., 2021) (Figure 1B). Expression can be detected throughout NPCs differentiation and into the pretubular aggregate, renal vesicle and S-shaped body stages of nephrogenesis (Figure 1B). Immunofluorescence analysis of E15.5 mouse kidneys further confirmed ETV4/5 protein localization in UB tips and developing nephrons with weak localization in the cap mesenchyme (Figure 1C). Next, and given the broad expression of *Etv4/5* across multiple components of the developing kidney, we investigated their compartment-specific roles.

ETV4/5 are critical for the development of the collecting duct system (Kuure et al., 2010; Riccio et al., 2016). Indeed, conditional ablation of *Etv5* in the ureteric bud using *Hoxb7-creEGFP* (Zhao et al., 2004) in an *Etv4*-null background recapitulated previously reported phenotypes (Lu et al., 2009), ranging from severely hypoplastic kidneys to complete renal agenesis (Figure 1D). Therefore, ablation of *Etv4/5* in the ureteric bud is sufficient to account for the phenotype of the double mutants. Whether ETV4/5 also function in NPCs and developing nephrons remained unclear, though. To address this, *Etv5* was conditionally ablated in NPCs and their derivatives using *Six2-creEGFP* (Kobayashi et al., 2008) in an *Etv4* null background. The resulting mutant kidneys were hypoplastic and cystic (Figure 1E) and the mutant pups rarely survive beyond the first postnatal week. These results identify an essential role of ETV4/5 in the NPCs and their derivatives during kidney development.

### 3.2 Absence of ETV4/5 triggers the premature differentiation of the NPCs

**FIGURE 2:**
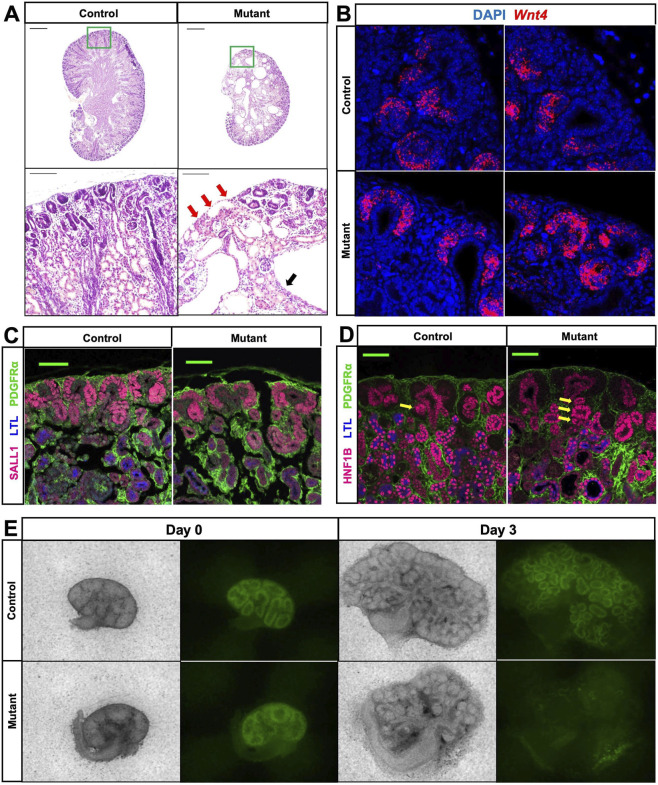
Disrupting *Etv4* and *Etv5* expression disturbs early nephrogenesis. **(A)** IHC of *Six2-cre* mutants reveals a disrupted nephrogenic zone that has areas of both active nephrogenesis (black arrow) and NPCs exhaustion (red arrows). Scale bar, 500 um and 100 um. **(B)** RNAscope staining showing ectopic *Wnt4* expression in the NPCs of *Etv4* and *Etv5* mutants. Immunofluorescent analysis at P0 of SALL1 **(C)** and HNF1B **(D)** indicate normal expression of the markers in both the NPC niche and early developing nephrons respectively. Yellow arrows highlight some early developing nephron structures. Scale bar, 100 um. **(E)** Live imaging assay of E12.5 *Six2-creEGFP* kidney explants after 3 days of culture showing both brightfield and GFP fluorescence. By day 3 of culture, mutant kidneys have almost completely exhausted the *Six2* ^*+*^ NPCs.

To further characterize this novel mutant phenotype, we analyzed the morphology of the developing kidney at birth/postnatal day 0 (P0). Hematoxylin-Eosin (H&E) staining revealed a marked reduction in kidney size and presence of cysts in the *Etv4/5* mutant kidneys (Figure 2A). In addition, the nephrogenic zone -the outer cortical region where renal progenitors reside-was interrupted in the mutant kidneys (Figure 2A, red arrows). To investigate premature NPC depletion, RNA-scope *in situ* hybridization was performed on E14.5 kidney sections to assess *Wnt4* expression. During normal kidney development, *Wnt4* marks early differentiation, being expressed in the pretubular aggregates where it is required for nephron differentiation (Stark et al., 1994). In *Etv4/5* mutants, ectopic expression of *Wnt4* was detected in the cap mesenchyme throughout the nephrogenic zone (Figure 2B), indicating premature NPC differentiation. When examined at P0, surviving NPC niches maintained SALL1 expression (Figure 2C) while markers of early developing nephron structures like HNF1B were expressed in a pattern comparable to control samples (Figure 2D)- indicating a competency of the mutant NPC niche to progress into early nephrogenesis. Of note, while ureteric buds in control kidneys induce small numbers of developing nephrons at a time, larger clusters of early developing nephron structures were seen below mutant ureteric bud tips (yellow arrows, Figure 2D). Consistent with this, *ex vivo* culture of E12.5 kidney explants revealed progressive loss of the nephrogenic zone. Live imaging of Six2-GFP–expressing explants demonstrated near-complete depletion of Six2^+^ NPCs in mutant kidneys after 3 days in culture compared with littermate controls (Figure 2E and Supplementary movie 1). Interestingly, progenitor loss appears to happen at a higher rate in cultured explants than *in vivo*. Six2^+^ cells are nearly completely absent after 3 days in culture while newborns often have small remaining patches of intact nephrogenic zone, suggesting that progenitor loss in *Etv4/5* mutants is exacerbated in culture. Together, these findings indicate that ETV4/5 are required to maintain the undifferentiated NPC population during kidney organogenesis.

**FIGURE 3:**
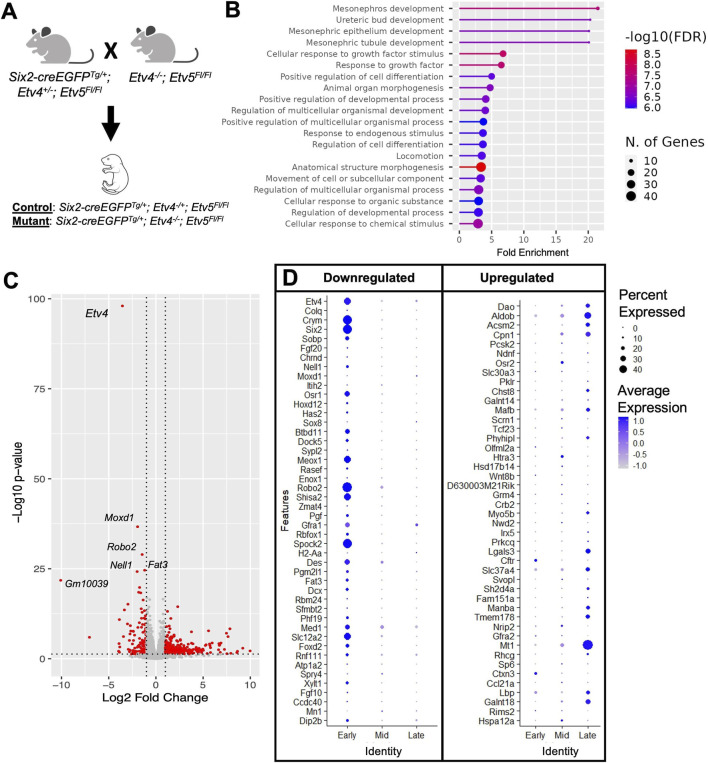
Disrupting the *Etv4* and *Etv5* expression alters the transcriptome. **(A)** Mating strategy for samples used in RNA-Seq analysis. **(B)** ShinyGO GO biological process analysis (Ge et al., 2020) for RNA-Seq results highlights the disruption to nephrogenesis, kidney development and pathways that are known to be related to ETV4 and ETV5 functions. **(C)** The volcano plot with a selection of the most significantly altered genes highlighting possible downstream targets for *Etv4* and *Etv5*. **(D)** Plot showing the top 50 most downregulated and upregulated genes in the *Six2-cre* mutants mapped against 3 distinct developing kidney scRNA-Seq clusters- “early”, “mid” and “late” (scRNA-Seq data from Combes et al., 2019). The downregulated genes in the *Six2-cre* mutants align with “early” developmental clusters while the upregulated genes align with the “late” nephron segment clusters.

To define the transcriptional consequences of ETV4/5 loss, we performed bulk RNA-seq on E14.5 kidneys (n = 4 mutants, n = 3 controls). Comparison of controls and mutant littermates (Figure 3A) identified pathways and genes affected by *Etv4/5* deficiency (see Supplementary Table S1 for a list of DEGs). 644 genes are differentially expressed in mutant kidneys (padj<0.05). Gene ontology analysis (Ge et al., 2020) revealed significant enrichment for terms related to nephrogenesis, collecting duct development, and kidney organogenesis (Figure 3B). Moreover, further analysis found several genes with significantly altered expression (Figure 3C), including Fibroblast Growth Factors *Fgf20*, *Fgf10* and *Fgf1* as well as *Spry4* and Itga8, all of which were downregulated in mutant kidneys. SPRY4 is a known downstream target of FGF signaling (Morgani et al., 2018) and ITGA8 is an adhesion molecule critical for epithelial-mesenchymal interactions in NPCs (Müller et al., 1997) that mediates MAPK-dependent NPC survival and differentiation (Ihermann-Hella et al., 2018). The differential expression of these genes in the ETV4/5 mutant is consistent with *Etv4/5* being downstream targets of FGF signaling in NPCs and suggest a possible feedback loop. To further interrogate the phenotype caused by ETV4/5 loss, we compared this RNAseq data with available scRNA-seq data from mouse embryonic kidneys (Combes et al., 2019). The top 50 upregulated and downregulated genes from the bulk RNA-seq analysis were mapped onto cell populations within the developing nephron grouped as early, mid and late developing structures. Downregulated genes corresponded to early nephrogenic populations, including naïve and induced NPCs whereas upregulated genes were enriched for markers of differentiated nephron segments such as podocytes, proximal tubules, and distal tubules (Figure 3D). Together with the exhaustion of NPCs and ectopic *Wnt4* expression, these results support a critical role for ETV4/5 in preventing premature differentiation of NPCs during kidney development.

### 3.3 Ablating *Etv4/5* from NPCs causes cysts to form in the proximal tubule

**FIGURE 4:**
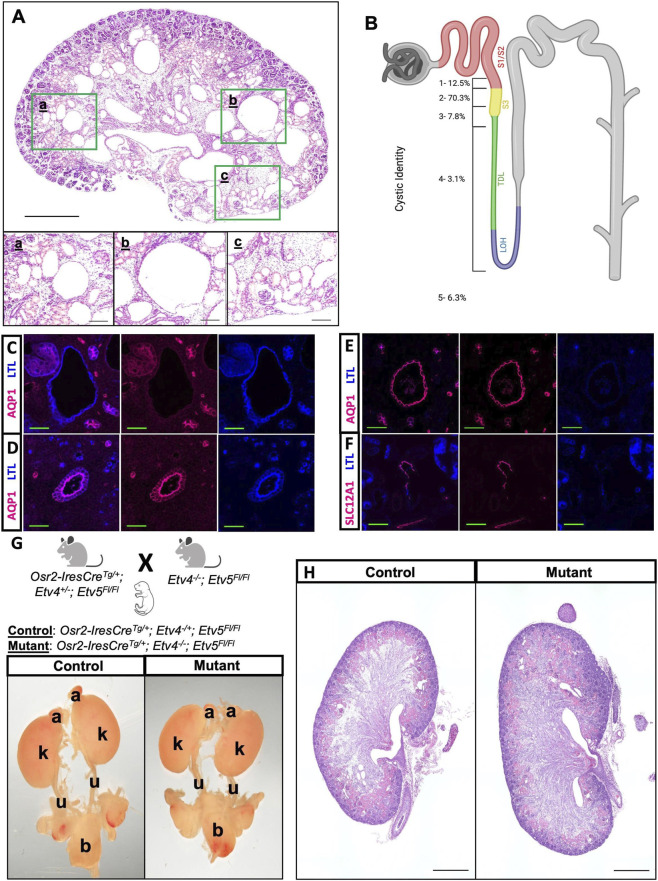
*Etv4* and *Etv5* mutant cysts have a distinct tubular identity. **(A)** H&E of P0 *Six2-cre* mutants show the varying morphology of cysts that develop. Scale bar, 500 um and 100 um. **(B)** Characterization of cysts into (5) categories based on immunofluorescent analysis (1 = S1/S1 identity, 2 = S3 identity, 3 = S3/tDL identity, 4 = tDL/LoH/hybrid cyst identity, 5 = unknown segment identity) (n = 5, 64 cysts). Diagram created in BioRender: Day, K. (2026) https://BioRender.com/ikmyjuo. **(C–F)** Examples of IF staining for cystic characterization of groups 1 **(C)**, 2 **(D)**, 3 **(E)** and 4 **(F)**. Scale bar, 50 um. **(G)** Mating strategy for an *Osr2-IresCre* line that removes ETV4 and ETV5 from the proximal developing nephron. Kidneys at P0 have normal morphology [a = adrenal gland, k = kidney, u = ureter, b = bladder]. **(H)** Periodic Acid-Schiff staining on P0 *Osr2-IresCre* kidney sections shows no significant phenotype in the mutants. Scale bar, 500 um.

**FIGURE 5:**
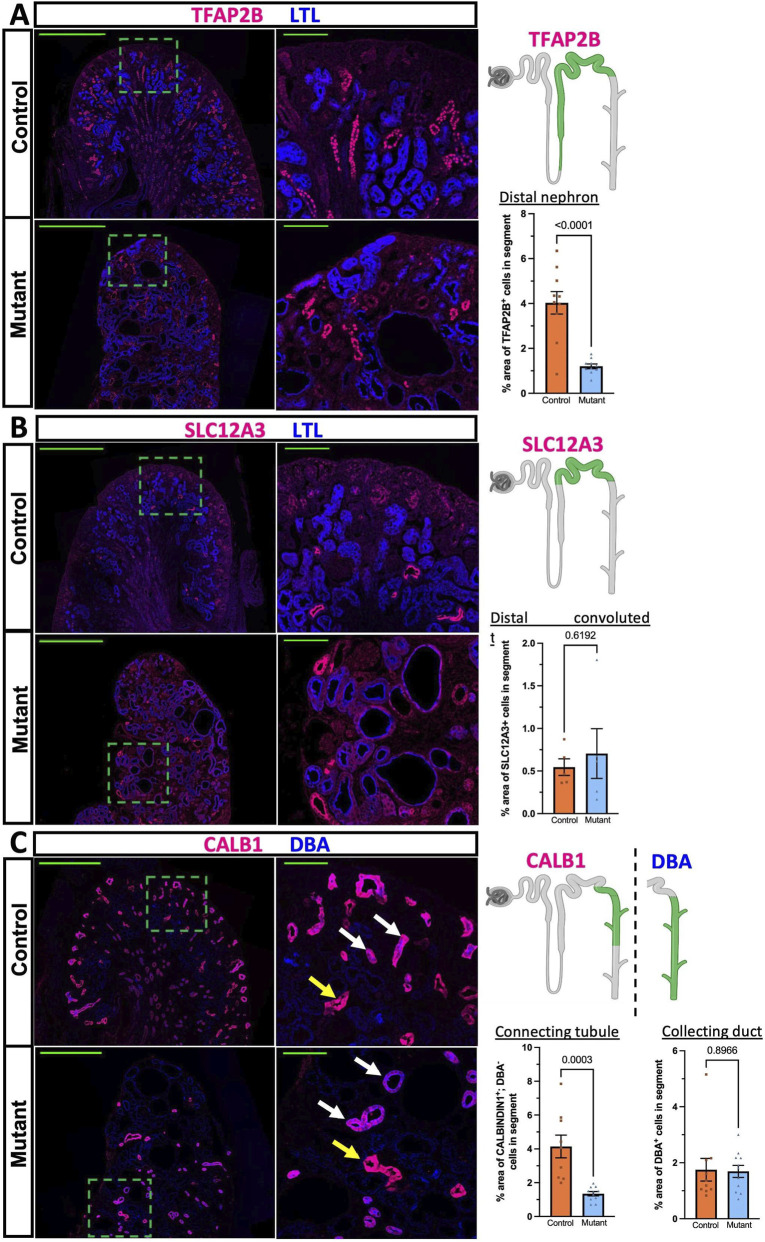
Distal tubule development is disrupted in Six2-Cre *Etv4* and *Etv5* mutants. Immunofluorescent analysis at P0 of distal nephron marker TFAP2B (n = 5C, 5M) **(A)**, distal convoluted tubule marker SLC12A3 (n = 5C, 5M) **(B)**, connecting tubule/collecting tubule marker CALBINDIN1 (n = 5C, 6M) and collecting duct marker DBA (n = 5C, 6M) **(C)** with their corresponding quantification for % of the section area that is positive for the relevant marker. Scale bar, 500um and 100um. Analysis of these three distal markers indicate that the connecting tubules of the P0 *Six2-cre* mutants are being affected by the loss of *Etv4* and *Etv5*. The quantification for the connecting tubules **(C)** is shown as the % area of cells that were CALB1^+^; DBA^−^ to isolate the CALB1 staining that is specific to the connecting tubule (yellow arrow) from CALB1 collecting duct staining (white arrows).

In addition to nephron progenitor cell depletion, we also observed significant cystic disease in newborn mutant kidneys. Histological analysis of P0 mutant kidneys revealed heterogeneous cyst and cystic epithelial morphologies within individual kidneys (Figure 4A). To define the segmental origin of these cysts, immunofluorescence analysis was performed at P0 using Lotus tetragonolobus lectin (LTL) to label proximal tubules, AQP1 to mark the S3 segment of the proximal tubule and the descending limb of the loop of Henle, and SLC12A1 to label the loop of Henle (Figure 4C). Quantification of 63 cysts from six mutant kidneys showed that the majority (70.3%) co-expressed LTL and AQP1 (Figure 4D), while 12.5% were LTL^+^/AQP1^-^ (Figure 4C) and 7.8% were AQP1^+^/LTL^−^ (Figure 4E). A small subset of cysts (3.1%) exhibited hybrid identity, expressing both LTL and SLC12A1 in a non-overlapping pattern within the same cyst (Figure 4F). No cysts expressed distal nephron markers, including TFAP2B or SLC12A3 (Figures 5A,B). These findings indicate that cysts arising from loss of ETV4/5 in developing nephrons predominantly originate from the proximal tubule, most likely the S3 segment, with minimal contribution from distal nephron segments (Figure 4B). This preference for S3 proximal tubule identity remains in the postnatal period, as revealed by analysis of rare postnatal survivors where the identity of cysts is near-exclusively LTL^+^/AQP1^+^ at P17 (Supplementary Figure 2).

To determine whether cyst formation resulted from a direct requirement for ETV4/5 in the proximal tubule, *Etv5* was conditionally ablated using *Osr2-IREScre* (Lan et al., 2007) in an *Etv4*-null background. *Osr2-IREScre* induces recombination in the S-shaped body and targets all nephron segments except for the distal and connecting tubules (Deacon et al., 2019). In contrast to *Six2-creEGFP* mutants, these kidneys exhibited no gross morphological abnormalities or significant cyst formation at P0, as assessed by gross morphology and H&E staining (Figures 4G,H). Mutant animals were viable and displayed no renal malformations in adulthood. These results suggest that cyst formation in *Six2-creEGFP* mutants is not due to loss of ETV4/5 within the proximal nephron itself but is likely secondary to ETV4/5 deficiency in other nephron compartments or in nephron progenitor cells.

### 3.4 Ablation of Etv4/5 from NPCs compromises the connection between developing nephrons and the collecting duct system

We next used immunofluorescent staining analysis of P0 kidney sections to identify and quantify the presence of different nephron segments of *Six2-creEGFP* Etv4/5 mutants. Whole sections were imaged, quantified, and represented as a percentage of total section area. Thresholds were used to discount the area occupied by cysts in the mutant kidneys (Supplementary Figure 2). A significant decrease in TFAP2B abundance was observed when comparing mutant and control littermate kidneys (Figure 5A). Since TFAP2B is a broad marker that is expressed in the ureteric bud and across the distal nephron at P0 (Marneros, 2020; Naganuma et al., 2021), we then employed markers whose expression is more spatially restricted. SLC12A1 is expressed in the Loop of Henle (LoH) and SLC12A3 is a specific marker of the distal convoluted tubule. There were no significant differences between control and mutant kidneys stained with anti-SLC12A1 or anti-SLC12A3 antibodies (Supplementary Figures 1B, 5B, respectively). This left one portion of the distal nephron to interrogate: the connecting tubule. The connecting tubule is the distal-most segment that connects the NPC-derived nephron to the ureteric bud-derived collecting ducts. To selectively identify this segment, we employed a dual staining analysis of both CALBINDIN1 (CALB1) and Dolichos biflorus agglutinin (DBA). CALB1 is expressed in both the connecting tubule and the collecting duct at P0, and DBA labels exclusively the collecting duct. For quantification, only staining that was CALB1^+^ and DBA^−^ was included, demonstrating that the *Etv4/5* mutants had significantly decreased abundance of the connecting tubule marker (Figure 5C). These data point towards a role for *Etv4/5* in the proper development of the connecting tubule during nephrogenesis and suggest a defect in the connection of the nephrons with the collecting ducts.

**FIGURE 6:**
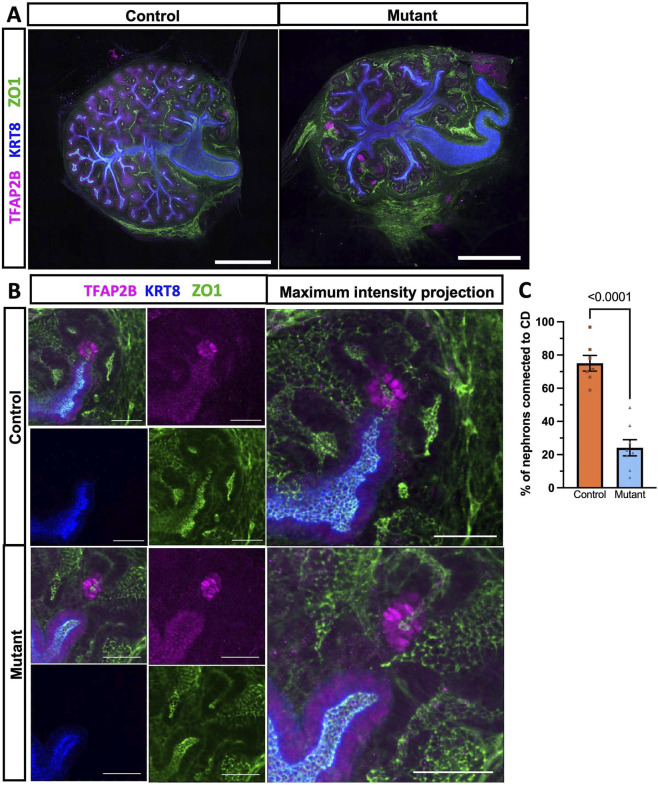
Developing nephrons in *Etv4* and *Etv5* mutants fail to connect to the collecting duct system. **(A)** Immunofluorescent analysis of E12.5 kidney explants after 3 days in culture shows altered branching and nephrogenic zones in the *Six2-cre* mutants. Scale bar, 500 um. **(B)** Representative immunofluorescence images of E12.5 *Six2-cre* explant mutant nephrons failing to connect the TFAP2B^+^ distal segment to the KRT8^+^ collecting duct. Scale bar, 50um. **(C)** Quantification of the immunofluorescence analysis reveals a significant decrease in the percentage of mutant nephrons that form proper connections to the collecting duct system (n = 7C, 8M).

Hence, we wanted to test if these connections are compromised in mutant kidneys. E12.5 kidney explants were cultured for 3 days in order to identify the early, developing connecting tubules during nephrogenesis (Figure 6A). By staining these explants for TFAB2B, KRT8 and ZO1 to label the early distal nephron segments, collecting ducts and epithelial lumens respectively, we were able to identify individual connections between developing nephrons and the collecting duct system (Figure 6B). Our data showed a significant decrease in the proportion of developing nephrons that connect to the collecting duct systems after 3 days in culture, 24% in mutants vs. 75% in control littermates (Figure 6C). These data demonstrate that *Etv4/5* ablation compromises the connection of the developing nephron to the collecting duct.

## 4 Discussion

Here we demonstrate that ETV4 and ETV5 play critical roles in the NPCs and, when ablated, a severe kidney phenotype ensues. This phenotype is three-fold: exhaustion of progenitors, proximal nephron cysts, and failure of the developing nephrons to connect to the collecting system.

The exhaustion of Six2^+^nephron progenitors, together with increased or ectopic differentiation of early nephrons, is consistent with a role for *Etv4/5* as downstream effectors of FGF signaling. Other known downstream targets of FGF signaling, like *Spry4* or *Dusp6* are downregulated in the *Etv4/5* mutants as well as *Fgf20*, *Fgf10* and *Fgf1.* This suggests a positive feedback loop where ETV4/5 regulate the expression of upstream effectors of FGFR signaling. Alternatively, and since these are all genes expressed in the NPCs, it could be a consequence of the overall reduction in these progenitors. The progenitor exhaustion phenotype has been reported in the *Fgf9* ^*+/−*^ *;Fgf20* ^*−/−*^ mutants (Barak et al., 2012) and in the NPC-specific mutants for *Fgfr1/2* that in addition also exhibit cystic tubules (Di Giovanni et al., 2015). This similarity of phenotypes is consistent with ETV4/5 acting as major components of FGF signaling in NPCs. However, the *Fgf9/Fgf20* double mutants (Barak et al., 2012) and the *Pax3-*driven *Fgfr1/2* mutants (Poladia et al., 2006) present renal agenesis at birth, a phenotype significantly more severe than that of the *Etv4/5* mutants presented here. We cannot discard that differences in genetic backgrounds may account for this divergence of phenotypes. In addition, a possible role of FGF signaling in the renal stroma is could also account for these differences. The *Pax3*-cre model drives recombination in the metanephric mesenchyme (Engleka et al., 2005) that comprises both the NPCs and the stromal progenitors. This could explain the more severe phenotype in the *Fgf9/Fgf20* double mutants and the *Pax3-*driven *Fgfr1/2* mutants, in which there would be no FGF signaling in NPCs nor in the stromal progenitors. Against this argument, though, ablation of *Fgfr1/2* in the stromal progenitors using Foxd1-cre has been mentioned, but not fully reported, to have normal kidneys (Walker et al., 2016). Therefore, it is more plausible that timing of recombination and/or differences in cre lines account for these different phenotypes. Indeed, the *Six2-creEGFP* line used to recombine *Fgfr1/2* ((Poladia et al., 2006) and *Etv4/5* (this study) has been shown to express cre mosaically in the NPCs (Surendran et al., 2010), with full recombination achieved by the S-shaped body. Interestingly, ablating *Mek1* and *Mek2*, the kinases that activate ERK1/2 downstream of receptor tyrosine kinases, from NPCs using the *Six2-creEGFP* line recapitulates the progenitor loss phenotype (Ihermann-Hella et al., 2018). The nephrons in this model, however, fail to develop further after induction, most likely due to failed FGF8 signaling in the early developing nephron (Grieshammer et al., 2005; Perantoni et al., 2005). This suggests that ETV4/5 are not the main effectors of MEK1/MEK2-mediated role of FGF8 in the developing nephron.

Proximal cystic tubules are the second phenotype we observe in the absence of *Etv4/5* in the NPCs. Our characterization of the cysts in the mutants identifies them as of proximal nephron origin suggesting that *Etv4/5* could have a specific role in this segment. In zebrafish, *etv5a* expression is restricted to the proximal tubule during nephrogenesis and *etv5a* morphants exhibit multiciliated cell depletion and edema (Marra and Wingert, 2016). However, when we ablate *Etv4/5* from the early proximal nephron in the mouse we fail to recapitulate the cystic phenotype. This could be explained by ETV4/5 being required in the very early nephron (pretubular aggregate or renal vesicle) before the *Osr2-cre* becomes active. Identifying this early role is extremely challenging, though. Mouse models of *Wnt4*-driven recombination have shown mixed success in ablating gene expression during early nephrogenesis. One report (Kanda et al., 2014) observes significant leaking into NPCs for the *Wnt4GC* model (Mugford et al., 2009) while others (Liu et al., 2018) report failure to eliminate the target proteins using the *Wnt4EGFP/cre* line (Shan et al., 2010), likely as a result of persistence of protein from NPCs. Indeed, to eliminate protein function in the early nephron structures, the remaining protein from NPCs needs to be degraded and the targeted alleles excised before the window of susceptibility passes. Since the progression from NPC induction to S-shape body formation in mice is estimated to last around 24 h (Lindstrom et al., 2018) it is possible that these models do not effectively target gene function in even earlier structures like the pretubular aggregate or the renal vesicle, when there is remaining protein from the NPC state.

An alternative explanation for the absence of cysts in the proximal nephron-specific model would be that the cysts are a consequence of aberrant nephron development caused by the increased/ectopic differentiation. Supporting this explanation, cystic kidneys are often observed in mouse mutants that present progenitor exhaustion and increased/ectopic differentiation, although those cysts are not usually characterized. For instance, NPC-specific *Sall1* and *Osr1* mutants have glomerular cysts and dilated tubules that are negative for LTL and Tamm Horsfall protein staining (Kanda et al., 2014) and epithelial cysts of unknown identity (Xu et al., 2014), respectively; and both the NPC-specific *Fgfr1/2* double mutant and the FRS2A binding domain mutant also present cysts that are LTL positive (Di Giovanni et al., 2015). Nevertheless, not all mutations that affect NPCs lead to cystic kidneys. The NPC-specific *Pax2* mutant does not develop cysts; instead, the nephron progenitors transdifferentiate into stroma (Naiman et al., 2017). *Lats1/2* ablation in NPCs has a very similar phenotype with no nephrons and increase in stromal cells (McNeill and Reginensi, 2017) and removing β-catenin signaling in NPCs results in hypoplastic kidneys but no cysts (Park et al., 2007). Therefore, defects in early nephron development caused by increased/ectopic differentiation, but not NPC maintenance, are a plausible explanation for the cystic phenotype we report here in the *Etv4/5* mutants.

Blind nephrons that fail to connect to the collecting duct system are the third phenotype observed in the *Etv4/5* mutants. It is not until the late S-shape body that the developing nephron establishes a continuous lumen with the collecting duct (Kao et al., 2012). Distal nephron precursors are required for this connection, and proximalization of the developing nephron by activation of the Notch intracellular domain (NICD) has been shown to compromise it (Kao et al., 2012). Therefore, the observed mutant phenotype suggests that ETV4/5 are required for the distal identity of the developing nephron. However, as mentioned above, we cannot discard the possibility that this phenotype is caused by non-segment specific overall defects in early nephron development. One attractive possibility is that unconnected nephrons are the ones that become cystic. However, no cysts are observed in the proximalized model (Kao et al., 2012). While this could be caused by the persistent expression of NICD in the proximalized nephrons, further studies will be needed to establish causality between the failed connection and the cystic phenotype in the absence of ETV4/5.

Pathogenic variants of *ETV4* have been reported in the literature (Chen et al., 2016; Kolvenbach et al., 2023) associated with congenital anomalies of the kidney and the ureteric tract (CAKUT) although these cases did not present with cystic disease. Given how critical ETV4/5 are for ureteric bud development, it is not surprising that mutations in these genes would result in renal agenesis or severe hypodysplasia in humans. On the other hand, the progenitor depletion, aborted connection and cystic phenotype we present here could provide additional insight into the cellular mechanisms driving CAKUT by other mutations. Does premature differentiation cause failed connection? Do failed nephrons become cystic? Answering these questions and identifying the mechanisms behind these phenotypes could be instrumental in understanding other CAKUT cases, most of which remain unexplained.

## Data Availability

The data discussed in this publication have been deposited in NCBI’s Gene Expression Omnibus (Edgar et al., 2002) and are accessible through GEO Series accession number GSE320548 (https://www.ncbi.nlm.nih.gov/geo/query/acc.cgi?acc=GSE320548).

## Supplementary material

SUPPLEMENTARY FIGURE S1 Proximal tubules and loop of Henle are present in *Etv4* and *Etv5* mutants. Immunofluorescent analysis of proximal segment markers AQP1(n = 5C, 6M), LTL (n = 11C, 7M) **(A)** and LoH/TAL segment marker SLC12A1 (n = 7C, 6M) **(B)** with their corresponding quantification for segment area as a percentage of segment area. Scale bar, 500um and 100um. Analysis indicates that the proximal and LoH segments are present in the *Six2-cre* mutants.

SUPPLEMENTARY FIGURE S2 Thresholding segment staining can be used to isolate CALBINDIN1^+^ connecting tubule staining. To isolate the connecting tubule, three thresholds were applied to the entire segment area **(Aa)**, DBA staining **(Ab)** and CALB1 staining **(Ac)** respectively. Then, using a custom GA3, any CALB1^+^ threshold from Ac that overlapped with DBA^+^ threshold from Ab was removed creating a fourth threshold **(Ad)**. Finally, after some manual clean up, this new threshold was used to quantify CALB1^+^, DBA^−^ collecting tubule in the kidney section. Scale bar, 500um. **(B)** The results of the thresholding analysis of CALBINDIN1 and DBA segment staining distinguishes between the CALB1^+^,DBA^+^ collecting duct (white arrows) and the CALB1^+^,DBA^−^ connecting tubule (yellow arrow). Scale bar, 100um.

SUPPLEMENTARY FIGURE S3 Tubular identity of *Six2-cre* mutant cysts remains segment specific in postnatal mutants. IF analysis of P17 *Six2-cre* mutants shows that cysts retain their S3 segment identity (LTL^+^, AQP1^+^) postnatally. Scale bar, 500um and 100um.

SUPPLEMENTARY VIDEO S1 Disrupting *Etv4* and *Etv5* expression disturbs *Six2* ^+^ NPC maintenance. Timelapse videos of E12.5 explant kidneys in brightfield and GFP views showing the loss of GFP^+^ NPC cells over the course of 3 days in culture. Scale bar, 500um.

SUPPLEMENTARY VIDEO S2 Developing nephrons in *Etv4* and *Etv5* mutants fail to connect to the collecting duct system. Video cycling through the Z-plane of E12.5 kidney explants after 3 days in culture shows faulty connections between the developing nephrons and collecting duct system (white arrow).
