# Supplementary figures and images for: Transcription factors ETV4 and ETV5 are required for nephron progenitor cell maintenance, distal nephron development and connection to the collecting system
Source: Front Cell Dev Biol. 2026 Jul 1;14:1820140. doi: 10.3389/fcell.2026.1820140 (PMC13370208; doi:10.3389/fcell.2026.1820140)

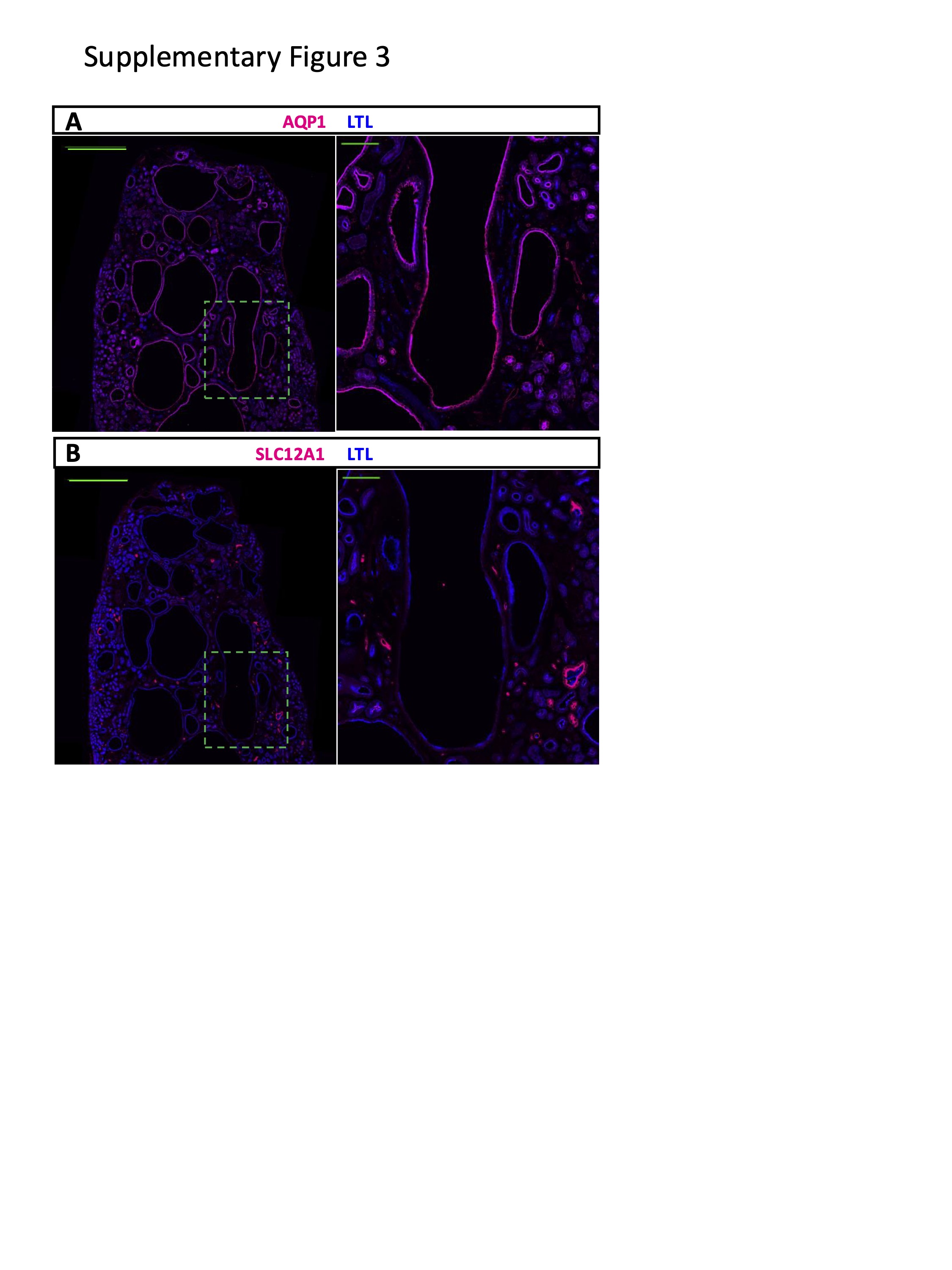

Supplement: Supplementary file 1 [file Image3.jpeg]

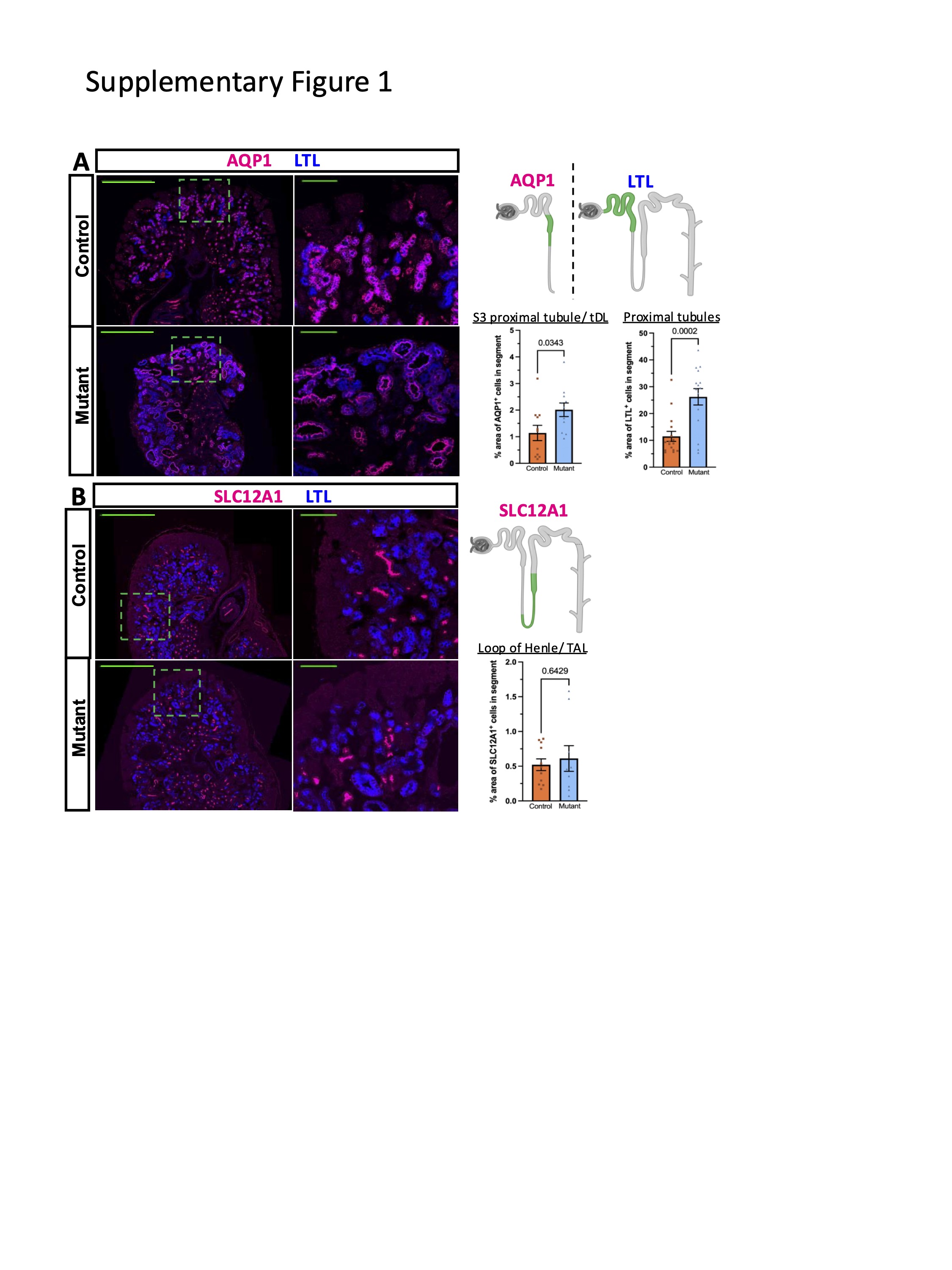

Supplement: Supplementary file 2 [file Image1.jpeg]

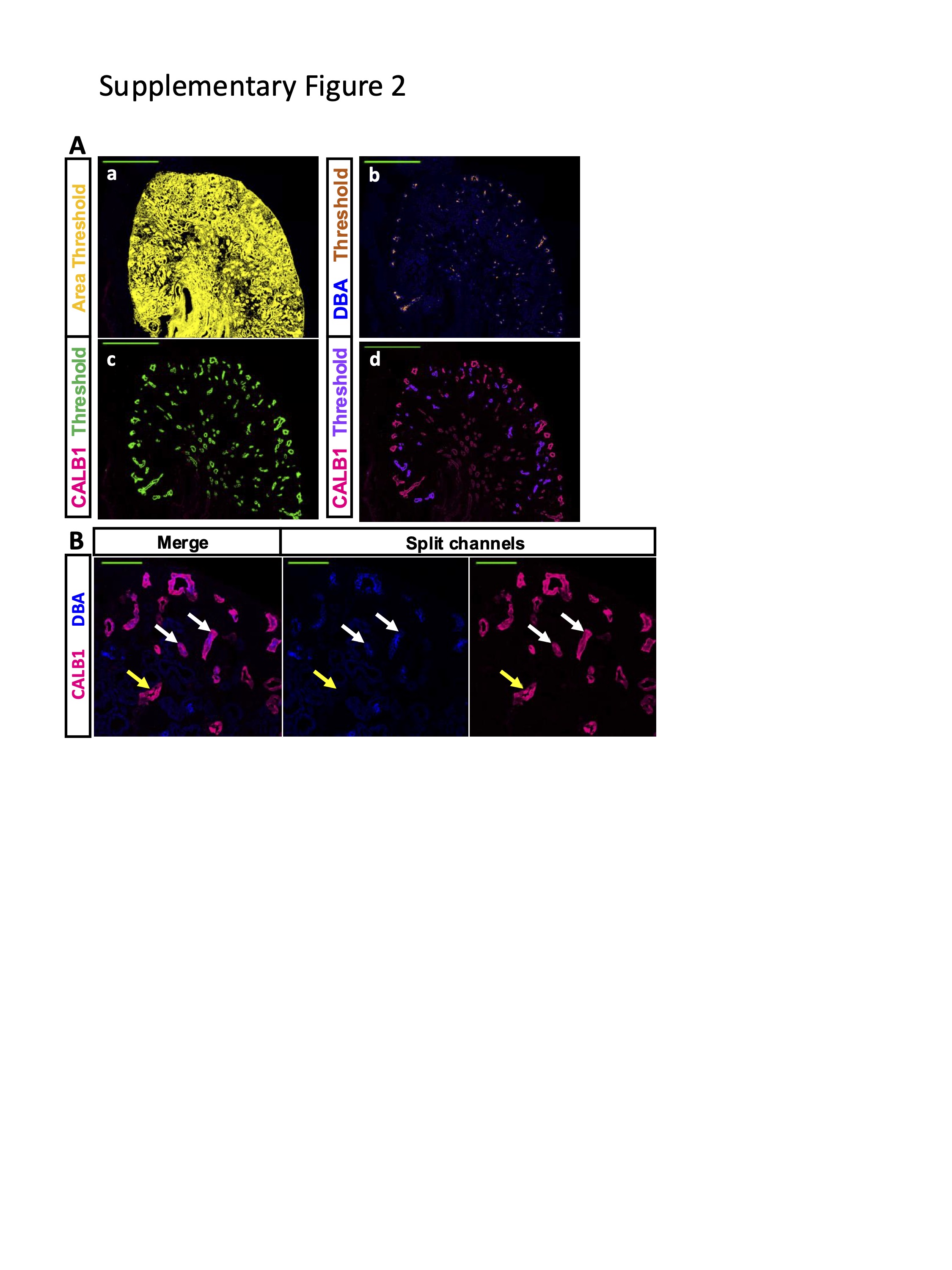

Supplement: Supplementary file 3 [file Image2.jpeg]
